# DNA G-segment bending is not the sole determinant of topology simplification by type II DNA topoisomerases

**DOI:** 10.1038/srep06158

**Published:** 2014-08-21

**Authors:** Neil H. Thomson, Sergio Santos, Lesley A. Mitchenall, Tanya Stuchinskaya, James A. Taylor, Anthony Maxwell

**Affiliations:** 1 Department of Oral Biology, School of Dentistry and Molecular and Nanoscale Physics Group, School of Physics and Astronomy, University of Leeds, Leeds, LS2 9JT, United Kingdom; 2 Department of Biological Chemistry, John Innes Centre Norwich Research Park, Norwich NR4 7UH, United Kingdom; 3 Current address: Laboratory for Energy and NanoScience (LENS), Institute Center for Future Energy (iFES), Masdar Institute of Science and Technology, Abu Dhabi, UAE.; 4 Current address: Intelligent Fingerprinting Ltd., NRP Innovation Centre, Colney Lane, Norwich, Norfolk NR4 7GJ, United Kingdom.; 5 Current address: Dept. Biochemistry, University of Bristol, Bristol BS8 1TD, United Kingdom.

## Abstract

DNA topoisomerases control the topology of DNA. Type II topoisomerases exhibit topology simplification, whereby products of their reactions are simplified beyond that expected based on thermodynamic equilibrium. The molecular basis for this process is unknown, although DNA bending has been implicated. To investigate the role of bending in topology simplification, the DNA bend angles of four enzymes of different types (IIA and IIB) were measured using atomic force microscopy (AFM). The enzymes tested were *Escherichia coli* topo IV and yeast topo II (type IIA enzymes that exhibit topology simplification), and *Methanosarcina mazei* topo VI and *Sulfolobus shibatae topo VI* (type IIB enzymes, which do not). Bend angles were measured using the manual tangent method from topographical AFM images taken with a novel amplitude-modulated imaging mode: small amplitude small set-point (SASS), which optimises resolution for a given AFM tip size and minimises tip convolution with the sample. This gave improved accuracy and reliability and revealed that all 4 topoisomerases bend DNA by a similar amount: ~120° between the DNA entering and exiting the enzyme complex. These data indicate that DNA bending alone is insufficient to explain topology simplification and that the ‘exit gate' may be an important determinant of this process.

DNA topoisomerases are enzymes responsible for maintaining and manipulating the topological state of DNA in all organisms^1^,^2^,^3^. These enzymes are required for vital processes such as DNA replication, transcription, recombination and chromatin remodelling. Topoisomerases can be classified into two types, I and II, depending upon whether their reactions involve transient cleavage of one (I) or both (II) strands of DNA. The enzymes can be further divided into sub-types (A and B) based on mechanistic and evolutionary considerations. Due to the important role played by topoisomerases in maintaining cell viability, they are attractive targets for chemotherapeutics^4^,^5^,^6^.

The basic mechanism of all type II topoisomerases involves the passage of one double-strand segment of DNA (the ‘T' or ‘transported' segment) through a double-stranded break in another (the ‘G' or ‘gate' segment) that is stabilised by covalent linkage (phosphotyrosine bonds) with the protein^2^,^3^. This strand-passage reaction is coupled to the binding and hydrolysis of ATP and accounts for their ability to relax and supercoil DNA and interconvert knotted/unknotted and catenated/decatenated forms^7^,^8^. All type II topos can relax DNA but DNA gyrase, present in all bacteria and some eukaryote organelles, can also catalyse DNA supercoiling. In the case of the gyrase supercoiling reaction, which is endergonic, the requirement for ATP is obvious. However, for the other type II enzymes, such as yeast topo II or bacterial topo IV, the requirement is less clear as their reactions are not obviously energy-requiring. However, some years ago it was shown that type II DNA topoisomerases can catalyse reactions ‘beyond thermodynamic equilibrium', that is the products of their reactions have steady-state levels of supercoiling, knotting or catenation below those seen at thermodynamic equilibrium^9^. This provided a potential explanation for the ATP requirement of non-supercoiling type II topoisomerases. However, subsequent work has suggested that the topology simplification process is unlikely to be of physiological significance and that the free energy requirements for simplification would account for less than 1% of that available from ATP^8^,^10^,^11^. It has alternatively been suggested that the free energy requirement is to control the separation of protein-protein interfaces in type II topoisomerases and thus prevent the formation of DNA double-strand breaks^10^.

Notwithstanding the significance of topology simplification, its mechanism is not currently clear. In the original paper^9^ the authors suggested that this was achieved by the enzymes tracking along the DNA; however, subsequent work has suggested that this is unlikely to be the case^8^. Another suggestion was kinetic proofreading involving two collisions of the enzyme with the DNA^12^, but recent single-molecule experiments have shown that this mechanism is unlikely^13^. A mechanism involving bending of the G segment bound to the enzyme, first suggested by Vologodskii et al.^14^, has received some support. The crystal structure of yeast topo II bound to DNA^15^ shows a profound bend of the G segment, which lends support to this suggestion; subsequent structures of bacterial topo IV and human topo IIβ also manifest G-segment bending^16^,^17^,^18^. AFM and FRET studies of three type IIA topoisomerases showed that while all three induced similar extents of G-segment bending, this did not correlate with the degree of topology simplification suggesting that G-segment bending may not be the sole determinant of topology simplification^19^.

All type IIA topoisomerases so far tested manifest some degree of topology simplification^8^,^9^,^19^, but *Methanosarcina mazei* topo VI, a type IIB enzyme, appears not to possess this property^8^,^20^. It follows therefore that there may be a mechanistic distinction between the type IIA and IIB enzymes implying that the latter do not show topology simplification. One possibility is that type IIB enzymes, such as *M. mazei* topo VI, do not stabilise a bent G segment. As there is currently no crystal structure of a type IIB enzyme bound to DNA, this remains an open question. Another possibility is that whereas type IIA enzymes possess three protein gates: the N-gate, the DNA gate and an exit gate, type IIB enzymes only possess two, i.e. they lack an exit gate. Recent work, including experiments with yeast topo II with an exit-gate deletion, supports this idea^21^.

In this paper we have used atomic force microscopy (AFM) to assess the extent of G-segment bending by type IIB topos in comparison with type IIA enzymes. Dynamic AFM (dAFM) imaging in amplitude modulation mode, often known as tapping, has been used previously to measure bend angles of other protein-DNA complexes, such as photolyase^22^, glycosylase^23^, integration host factor^24^, MutS^25^, centromeric DNA binding factor 3^26^, *E. coli* RNA polymerase^27^,^28^, three type IIA topoisomerases^29^ and others^30^ as well as the extent of wrapping of DNA around protein complexes such as DNA gyrase^31^ and RNA polymerase^32^. Here we utilised a recent development in dAFM imaging known as small amplitude small set-point (SASS), which optimises resolution for a given tip size and has ultra-low wear characteristics in ambient imaging, enabling high quality image data at higher throughput than conventional dynamic AFM methods^33^. In SASS, the tip scans with sub-nanometer oscillation amplitudes inside the ubiquitous nanoscale thick surface water films^33^,^34^ and close to the solid surface where the highly localized short-range forces are found^33^,^35^, simultaneously resulting in sufficiently small peak forces and low dissipation compatible with soft matter imaging^36^. The DNA bend angle study here illustrates an advantage of SASS imaging over conventional tapping mode.

## Results

### The application of SASS AFM imaging

**Figure 1:**
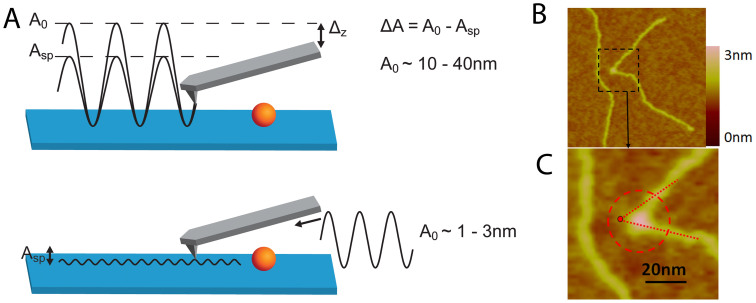
Comparing differences in operation between AFM imaging modes. A. Schematics of conventional Amplitude-Modulation AFM or Tapping Mode (top) versus Small amplitude small set-point (SASS) mode (bottom). B & C. Illustration of the reduced tip convolution in SASS mode as compared with typical tapping mode resolution. The AFM topographical image has been taken in SASS mode and the dashed red circle superimposed is the typical size the enzyme would appear in tapping mode, ~30 nm across. The straight dashed lines show where the tangents to the DNA backbone would be taken for a tapping mode image. One would expect these two tangents to intersect somewhere close to the centre of mass of the enzyme (as projected into two dimensions). The intersection here lies outside the topographical image envelope of the enzyme as measured by SASS mode. Looking at the SASS image we see that for this particular complex the acute bend angle is larger than would have been measured by tapping mode.

Conventional tapping-mode imaging is operated as follows. First, an appropriate free amplitude of the AFM cantilever off the surface is excited. Secondly, the tip is engaged to the surface with a given reduction in the amplitude (typically 5 to 30%) and used to topographically track the surface. As discussed in the Introduction, the downside of this approach is that the minimum distance of approach of the AFM tip to the surface is determined by the percent reduction in the free amplitude regardless of the interaction causing this reduction. In amplitude modulation, the frequency of the cantilever is free to shift in response to the conservative force interactions between tip and surface, which, at a set drive frequency of the cantilever, can cause the force-sensing cantilever to halt at a position where the imaging tip is on average not close enough to the sample surface to optimise resolution^33^,^34^,^35^. SASS mode overcomes this limitation by first engaging the tip to the surface with the minimal excitation amplitude possible. Second, the excitation of the cantilever is increased until a stable image is formed. By putting the tip in close proximity to the sample's surface with an initial small amplitude, the tip will be, on average, closer to the sample's surface in SASS than in conventional tapping mode. The difference in operation between tapping mode and SASS mode is schematically represented in Figure 1.

SASS mode imaging was carried out by engaging the tip to the surface with small free amplitudes of A_0_ ≈ 1 nm and maximum set-point amplitude ratios A/A_0_ ≈ 0.95. Once the surface is located, the set-point is reduced to A ≈ 0.1–0.3 nm. The excitation or drive amplitude is then slowly increased to increase the free amplitude A_0_ until the minimum required to achieve stable, high quality imaging is reached (i.e. A_0_ ≈ 2–5 nm in our experiments). For the Nanoscope Multimode AFM, typical values are A ≈ 0.01 V and A_0_ ≈ 0.2 V. For the AC160TS cantilevers this corresponds to A ≈ 0.15 nm and A_0_ ≈ 3 nm. The low-wear characteristics and high stability of SASS mode enabled data for each topoisomerase to be acquired with the same AFM tip. This gives high confidence that the size of the tip remained the same during the course of the data acquisition and no increase in the apparent width of the DNA or protein was observed during data acquisition. The low tip wear, even after hours of imaging in SASS, was further corroborated using an *in situ* tip-sizing technique which utilises the critical amplitude (A_c_) method^37^. In this method, the critical free amplitude A_c_ at which attractive to repulsive force transitions are observed provides information about the tip radius (R) via a functional relationship that was found experimentally for AC160TS cantilevers on mica, i.e. R ≈ 4.75A_c_^1.1^. Arrays of SASS topography and phase images were acquired by employing the autoscan mode and selecting a 10 by 10 matrix that allowed scanning 100 μm^2^ of surface area (100 times 1 × 1 μm scans).

### SASS-AFM imaging of type II topoisomerase–DNA complexes

Figure 1 illustrates schematically the difference between conventional AM AFM imaging modes such as Tapping Mode and the SASS mode we have previously developed and utilised in this application^33^. In essence, the reduced amount of energy stored in the cantilever when operating in SASS preserves the sharpness of the AFM tip. In ambient conditions the tip is engaged to the sample and remains inside the water condensate present between tip and sample^33^,^34^. This enables greatly reduced amplitudes for stable imaging with ultra-low tip wear and brings the tip, on average, closer to the sample thereby increasing resolution^33^,^38^,^39^.

Figure 1C demonstrates the increase in resolution SASS can give over typical Tapping-Mode imaging. The dashed circular outline is the size that a globular protein with a molecular weight comparable to the topoisomerases studied here will typically appear in conventional tapping mode repulsive regime imaging^31^. The diameter of ~30 nm is consistent with data for *E. coli* topo IV and yeast topo II in a previous tapping mode AFM study of DNA bending by type IIA topoisomerases^29^. The SASS topography image shows that the lateral tip convolution with *E. coli* topo IV in this case, is considerably smaller, improving the accuracy of the DNA bend angle determination.

**Figure 2:**
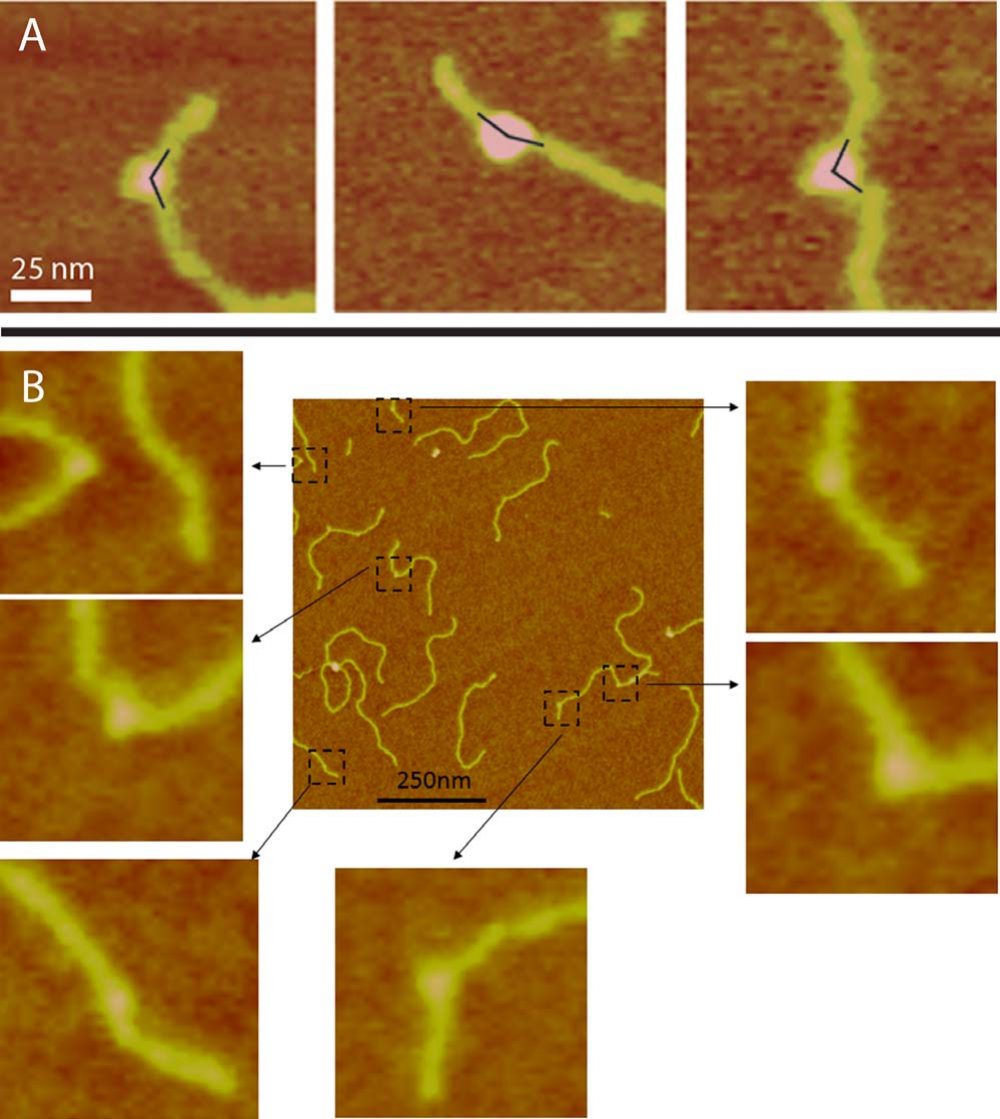
Measurement of bend angles. A. Examples of how the bend angles were measured from the intersection of tangents defined for the incoming and outgoing DNA strands. The measured angle was taken as the angle between the incoming and outgoing DNA strands, i.e. <180°. B. An example of a field of *E. coli* topo VI molecules and the corresponding high magnification software zooms of DNA bending. The enzymes are readily distinguished in the false colour scale image as having a higher height than the DNA.

Figure 2 shows typical SASS images acquired at 1 × 1 μm for *M. mazei* topo VI, with corresponding software zooms used to measure the bend angle. In one field of view there are several molecules with a large range of apparent DNA bend angles. All the topoisomerases studied yielded comparable AFM images in terms of molecular distributions and resolution, the enzyme diameters being ~15 nm (see Supplementary Material).

**Figure 3:**
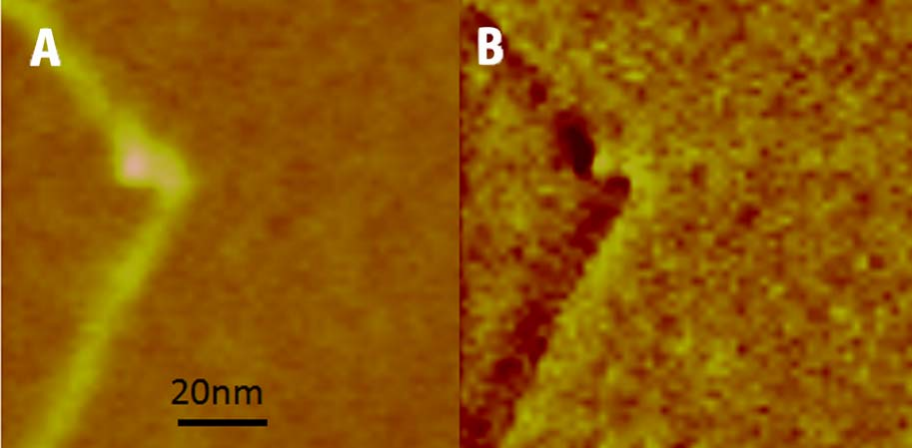
Demonstration of the increased resolution of SASS. The increased resolution in SASS enables us to discriminate complexes where the DNA bend does not appear to correspond exactly to the position of the topo protein. A. Topographical image. B. Phase contrast image in SASS mode.

The increased resolution in SASS enables us to more readily distinguish between complexes where the DNA bend occurs within the enzyme-DNA complex and those where a DNA bend may occur apparently just outside the topoisomerase (Figure 3). The phase contrast image (Figure 3B) for the dynamic SASS mode shows the central part of the enzyme clearly and correlates with the topographical data (Figure 3A). Phase contrast data were acquired simultaneously for all samples and were used to give improved confidence for the bend angle assay by AFM. Complexes such as those shown in Figure 3 were rejected for bend angle analysis. This highlights the validity of high resolution modes, and more specifically the validity of the SASS mode in ambient AFM analysis, over conventional tapping modes for making DNA bend angle measurements for protein complexes.

### Bend angle analysis of type II topoisomerases–DNA complexes

**Figure 4:**
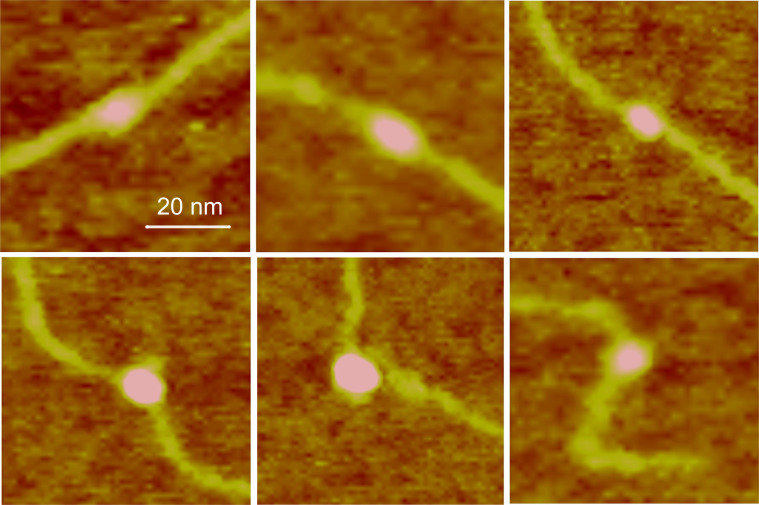
Examples of *M. mazei* topo VI images. *M. mazei* topo VI samples show two classes of complex: unbent (top row) and bent (bottom row). Data were similar for *E. coli* topo IV, *S. shibatae* topo VI and yeast topo II (see Supplementary Material).

**Figure 5:**
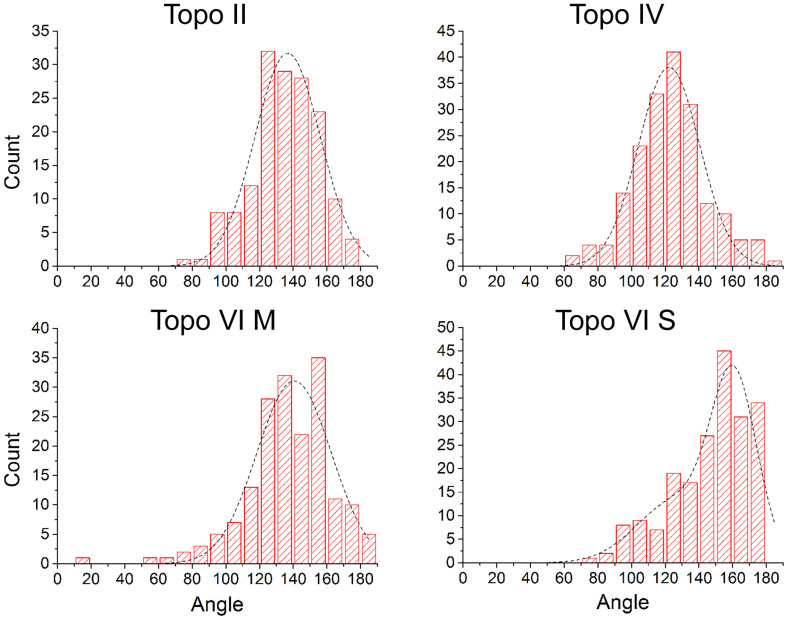
Full bend angle distributions. The full distributions for all four enzymes gave an obtuse angle for the defined bend angle inside the two DNA arms. The distributions decayed sharply towards the straight through value of 180°. All the enzymes, except *S. shibatae* topo VI were fitted to a single Gaussian, whereas this was fitted with a sum of two folded Gaussians. R^2^-values were: topo II = 0.95; topo IV = 0.96; topo VI M = 0.89; topo VI S = 0.87. (M), enzyme from *M. mazei*; (S), enzyme from *S. shibatae*.

Measurement and analysis of complexes for each topoisomerase studied revealed that a significant proportion of the complexes did not appear to bend the DNA (Figure 4, top row). Distinct DNA angles were measured for the majority of the molecules for all topo enzymes (~60–70%), however, and were detected with roughly equal abundance for each of the topoisomerases tested, except *S. shibatae* topo VI. Pooling data for all of the complexes for a given enzyme yielded the DNA bend angle distributions in Figure 5. Fitting of the distributions with Gaussians yielded values for the mean and standard deviation of the bend angle (Table 1). All enzymes were fitted with single peak Gaussians, except *S. shibatae* topo VI, which was fitted with a sum of two folded Gaussians. It was found that the other enzymes did not benefit from fitting with folded Gaussians as previously described^40^, because the proportion of complexes yielding straight-through conformations (i.e. no bend = 180°) was too low. The half-widths (twice the standard deviation) ranged from 36.4° to 47.6° as fitted with a normal distribution. Bend angle distributions for other protein-DNA complexes imaged using AFM tapping mode typically yield half-widths of ~90 to 120° for protein complexes with molecular weights of 380 kDa or above^25^,^26^,^27^,^28^,^32^ again demonstrating that SASS mode gives a resolution advantage. For comparison, the molecular weights of the type II topoisomerases studied here are: *M. mazei* topo VI, 220 kDa; *S. shibatae* topo VI, 211 kDa; yeast topo II, 328 kDa; *E. coli* topo IV, 306 kDa.

**Figure 6:**
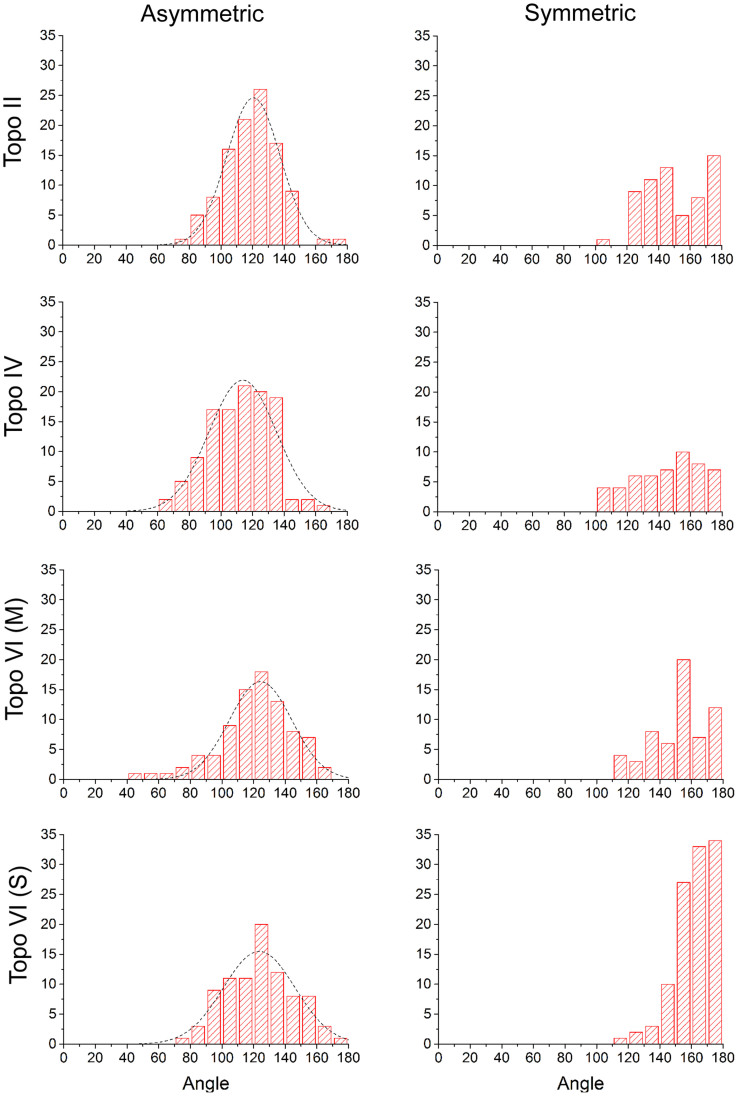
All type II topos bend linear DNA by a similar amount (~120°). The asymmetric complex distributions all fit to a single Gaussian with bend angles summarised in Table I. R^2^-values were: topo II = 0.98; topo IV = 0.92; topo VI M = 0.96; topo VI S = 0.91. The ratio of bent to unbent complexes is in the range of 0.7 to 2.2 for the conditions of the AFM experiments depending on the enzyme. (M), enzyme from *M. mazei*; (S), enzyme from *S. shibatae*.

It was observed that a significant fraction of the complexes appeared to exhibit no significant bend in the DNA (see Supplementary Material). It was assumed that this might be caused either by a non-preferential orientation of the complexes on the mica surface and/or because of two different modes of binding to the DNA, although only *S. shibatae* showed a clear bimodal bend angle distribution. To refine the bend angle determination, the data were split into two groups: symmetric (unbent) and asymmetric (bent) complexes. This was carried out via visual inspection of the images, where two independent operators gave similar outcomes. Figure S1 shows the outcome of splitting the data set for *E. coli* topo VI into two distributions. The half-width of the histogram for the asymmetric (bent) complexes is reduced by 6.2°. The data sets for all four topo enzymes were treated in this way and all were fitted by a single peak Gaussian, giving similar mean bend angles of ~120° (Table 1 and Figure 6). The ratio of the asymmetric to symmetric complexes is in a similar range for all enzymes, varying from 0.7 to 2.2 (see Table I), but *S. shibatae* topo VI has the lowest proportion of unbent complexes by a factor of 2 or more. This reflects the higher proportion of enzymes bound to the DNA without exhibiting a bend (Fig. S5) and could be a reflection of the thermophilic nature of this species. Consequently, this was the only enzyme that required a double Gaussian fitting to the full distribution.

### Bend angle correlation with protein molecular weights

**Figure 7:**
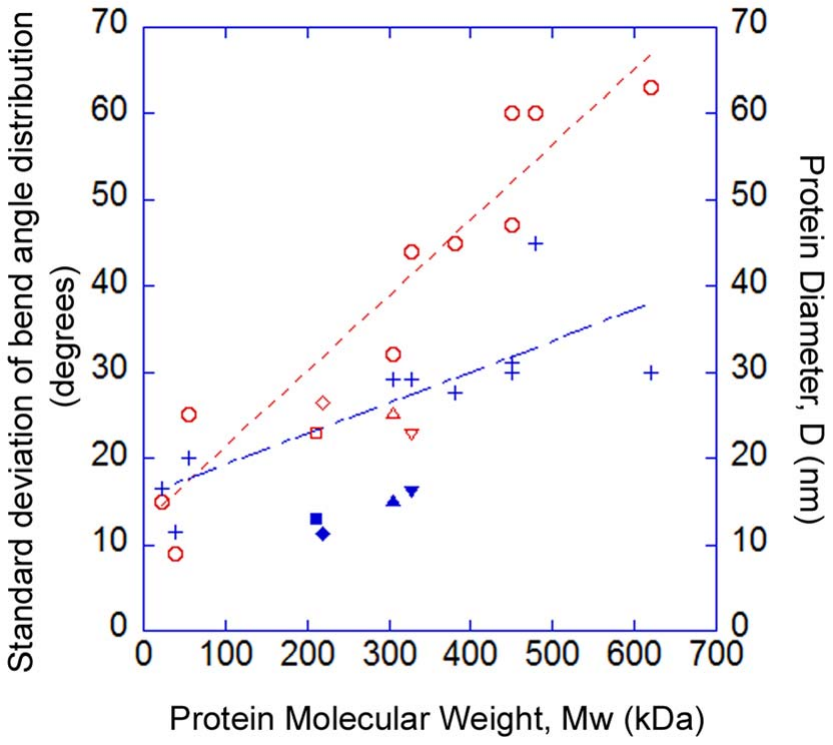
Correlation between protein molecular weight and bend angle. Correlations of the standard deviation of the bend angle distribution (red line and open circle markers) and the protein diameter, D, as measured by AFM (blue line and cross markers) versus protein molecular weight. Measurements made in tapping mode on other DNA-binding proteins (open circle and cross markers) show that the uncertainty of measuring the bend angle increases with molecular weight with a linear correlation. SASS measurements on type II topoisomerases for protein diameter and bend angle standard deviation (solid and open markers respectively: square, diamond, triangle, inverted triangle) do not show this correlation and demonstrate improved resolution as the markers lie at smaller values than the correlations from other published AFM studies. The standard deviation of the topo enzyme distributions plotted here were taken from the full bend angle distributions. The previous measurements of *E. coli* topo IV and yeast topo II^29^ are at molecular weights of 306 and 328 kDa respectively. Symbols: Squares – *S. shibatae* topo VI; diamonds – *M. mazei* topo VI; triangles – *E. coli* topo IV; inverted triangles – yeast topo II.

Analysis of previously published work on the measurement of DNA bend angles, formed by protein complexes by conventional tapping mode AFM, reveals a correlation between the width of the distribution of the measured bend angle and the size (molecular weight) of the protein. It has been shown before that there is a linear correlation between protein molecular weight and molecular volumes as measured by AFM^41^. Since the effective height of nanoscale objects, such as proteins, is related to the tip size^38^, we predicted a linear relationship between the measured protein diameter and molecular weight. Figure 7 shows a plot of the standard deviation of the bend angle distribution (red line) and the diameter of the protein as measured by tapping-mode AFM (blue line) against protein molecular weight. These values have been extracted from previously published data^22^,^23^,^24^,^25^,^26^,^27^,^28^,^29^,^32^ and relate to the following protein-DNA complexes: MutS, glycosylase, photolyase, centromeric DNA binding factor 3, integrin host factor, RNA polymerase complexes, and *E. coli* topoisomerase IV and yeast topoisomerase II. The relationship between these two parameters and molecular weight are linear with R^2^-values of 0.95 and 0.81, respectively. For larger protein complexes, the physical convolution with the AFM tip increases giving an increased measured diameter and as a consequence a larger spread in bend angle values. This is a direct consequence of a larger protein occluding the AFM tip from detecting the DNA backbone close to the protein complex. This concept has been exemplified previously in a study of DNA wrapping around DNA gyrase^31^. The values of the bend angles from this study of the four topoisomerases are plotted on the same graph and both the measured protein diameter and standard deviation of the bend angle distribution lie well below the values that the other studies would predict (Figure 7). This confirms that SASS mode gives improved overall resolution by reducing the tip convolution between the tip and sample by keeping the cantilever on average closer to the surface as compared with conventional dAFM modes.

## Discussion

### DNA bend angle measurements by AFM

Although AFM acquires 3D topographical information of the sample over which the AFM tip scans, for DNA bend angle measurements the complexes are effectively projected into two dimensions. For all topoisomerases tested, between 31 and 58% appeared as unbent or have a very small bend in the DNA (i.e. close to 180°) depending upon the enzyme. It could be that there are two modes of binding and these enzymes have not bent the DNA or alternatively the plane of the bend could be lying in the vertical plane (relative to the mica surface) and is therefore occluded by the protein and undetectable by the AFM tip. As mentioned already, bend angles of other large protein-DNA complexes using AFM imaging in tapping mode typically yields a half-width of ~90-120°^25^,^26^,^27^,^28^,^32^. This broadening of the distribution could be a combination of intrinsic flexibility in the protein as well as variations in complex binding to the 2D mica surface. The weighting of these two effects in each case will depend on the relative strength of binding of the DNA and the enzyme to the mica surface.

The separation of complexes into symmetric (unbent) and asymmetric (bent) was carried out via visual inspection by two independent operators. Along with the higher resolution afforded by the SASS imaging mode, this separation of complexes with apparently different behaviour yielded reliable bend angles with smaller statistical error. One could argue that selecting complexes for two separate classes is somewhat arbitrary; however, we argue that it is consistent with the intrinsic flexibility of the DNA from polymer chain models. Figure 6 shows that the two separated distributions overlap for angles between 120 to 160°. In this region, the probability of determining a complex as being bent or unbent is close to 50%. Taking the DNA to be a worm-like chain polymer, the relationship between the average bend angle, θ, over a contour length, L, in two dimensions is: 
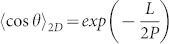
where P is the persistence length of dsDNA^42^. We can take the size of the topoisomerase protein in the SASS images and calculate the expected change in DNA angle from the intrinsic flexibility of the DNA over the length of the DNA that is occluded by the protein. The persistence length of dsDNA has been previously measured as 53 nm^43^, and for the typical size of the topo proteins by SASS (Fig. 7) we take L = 15 nm, then θ = 29.8°, so the average angular deviation (180-θ) = 150.2°. We can see therefore, that we would expect DNA to deviate away from the straight-through value of 180° to a mean value of ~150° over a 15 nm contour length. This value fits very well with the centres of the symmetric distributions in Fig. 6 and supports our method to separate complexes into two classes to determine a finite bend angle. In this study, we have defined the bend angle using 25 nm of DNA contour length (including the 15 nm occluded by the protein) which equates to about half the persistence length of DNA under the ionic strength of our deposition buffer at 16 mM^44^. Previous studies using tapping mode AFM have needed to use a longer length of the DNA to define the contour length due to lower resolution and larger tip convolution. For example, the previous study on type IIA topoisomerases used between 1.4 to 2.6 times the persistence length of DNA^29^.

In previous work, studying the wrapping of DNA around DNA gyrase, a significant proportion of the complexes remained with the DNA unwrapped (in the absence of nucleotide)^31^. This was attributed to an equilibrium between the DNA being wrapped and bound around the DNA gyrase enzyme and the DNA being more strongly attracted to the mica support surface and pulled off the gyrase surface. More recent work using magnetic tweezers has revealed that the wrapping of DNA around DNA gyrase is a very dynamic process where the DNA can be unwrapped for a significant proportion of time, even in the absence of a competing surface^45^. It is likely that DNA bend angle measurements of protein complexes on mica are affected in a similar way, and possibly to a greater extent. The relative proportion of complexes that are unbent in this study are in a similar range for each topo enzyme and served as a rationale for splitting the data sets into symmetric and asymmetric complexes to more reliably determine a distinct and meaningful DNA bend angle.

### SASS mode imaging enhances reliability of data

Even when Tapping-Mode imaging in ambient conditions is operated with careful choice of operational parameters, e.g. with a high set-point amplitude and relatively small free amplitude, it is usually impossible to eradicate wearing of the AFM tip over the imaging time-scale of hours. The AFM tip gradually broadens as imaging proceeds changing the fidelity of the data during the experiment and requires that more than one AFM tip is used to acquire a single data set. The benefits of SASS mode^33^ mean that data on single molecule complexes on hard background surfaces, such as mica, can be obtained with sufficient statistics where the resolution of the imaging is not degraded during the experiment. Importantly, this implies that data sets can also be compared with tips of similar size during all scans. If the end of the tip varies significantly during scans, statistics would be compromised since true variations in height, width, angle, etc. of the molecules would couple with variations in measurements due to variations in tip size R. SASS also optimises the available resolution for a given tip size. Both of these advantages enabled us to acquire bend angle distributions at significantly smaller half-widths than has been previously achieved.

Here we determine the bend angle of different topoisomerases by the manual tangent method. There are other methods to determine bend angle from AFM images^29^,^40^,^46^, including automated protocols for assigning the protein centre and the associated DNA tangents. Another method was introduced that utilised only the measurement of the straight line end-to-end distance (EED) between the ends of the DNA, without considering the protein complex directly^46^. It had been shown previously that under equilibrating conditions, the EED distance can be modelled using worm-like chain polymer statistics for DNA with an intrinsic bend^32^. This method was extended to protein-DNA complexes, by utilising the full EED distribution rather than just the mean squared EED^46^. It was shown to be effective for smaller DNA binding proteins where the resolution of standard tapping mode AFM makes it difficult to resolve the protein bound to the DNA. All these methods were utilised more recently to measure the bend angles of type IIA topoisomerases and compared with a FRET method^29^. This study found that *E. coli* topo IV, yeast topo II and human topo IIα all bent DNA by similar amount and are in broad agreement with our measurements. In future work, it would be interesting to compare these methods on images taken using SASS mode with its intrinsically higher resolution. It is important to note however, that the EED method assumes that there is only one sharp intrinsic bend in the DNA molecule caused by a protein. The data taken in this study show as many as 30% of complexes have sharp intrinsic bends that lie outside the protein complex (see Supplementary Material). Sometimes these bends lie close enough to the protein that in a tapping mode image they would be incorporated into the bend angle measurement because it would be occluded from the AFM tip. We believe that these bends may be a consequence of the protein affecting the equilibration of the DNA into two dimensions on the mica surface as previously discussed. It has been demonstrated by AFM, however, that DNA may not follow the worm-like chain model at short length scales^47^. This raises questions about the validity of other bend angle methods, such as EED, when the resolution in the AFM is not optimal.

### Type II topoisomerases bend DNA by a similar amount

In addition to demonstrating the advantages of SASS mode, this work has investigated the question of whether topology simplification by type II DNA topoisomerases is determined by the bending of the DNA G segment. Previous work^19^ showed that three type IIA topos, which show different degrees of topology simplification, bend DNA to a similar degree. Our data now shows that two type IIB topos, *M. mazei* and *S. shibatae* topo VI, which do not show topology simplification, bend DNA to a similar extent as two type IIA enzymes. Taken together these results strongly suggest that DNA G-segment bending is not the sole determinant of topology simplification by type II DNA topoisomerases. In that case we would suggest that another structural difference between type IIA and IIB enzymes must account for this effect. We propose that the exit gate (‘C-gate'), which is present in type IIA topos but absent in type IIB enzymes, most likely contributes to the topology-simplification phenomenon. We can envisage that its presence in type IIA enzymes might inhibit reverse strand passage having the effect of skewing the distribution of the products of relaxation, catenation/decatenation and knotting/unknotting reactions. Recent experiments^21^ support the notion of ‘backtracking' of the T segment in topo II enzymes, a concept originally described as ‘on-enzyme equilibrium' with DNA gyrase^48^. Construction of a yeast topo II mutant with an exit-gate deletion generates an enzyme unable to carry out topology simplification^21^, strongly supporting the idea that topology simplification is dependent on exit-gate integrity. However, the authors report that this deletion mutant is about two orders of magnitude less active that the wild-type enzyme and that these data must be interpreted with caution. Indeed our attempts to construct such mutants in *E. coli* topo IV have resulted in enzymes with little or no activity. Notwithstanding this, the data in this paper, and previously published work^8^,^13^,^19^,^21^, strongly support the idea that the DNA exit gate is an important determinant of topology simplification by type IIA topoisomerases.

## Methods

### Enzymes and DNA

*E. coli* topo IV and *M. mazei* topo VI were prepared as described previously^20^,^49^. Yeast topo II and samples of *M. mazei* topo VI were gifts from J.M. Berger (Univ California, Berkeley, USA). *S. shibatae* topo VI was a gift from P. Forterre (Institute Pasteur, Paris, France). All stocks of enzymes were in 50 mM Tris (pH 7.5), 100 mM NaCl, 10% glycerol and 2-mercaptoethanol. Enzyme assays were carried out as described previously^8^,^50^. A 1070 bp fragment of plasmid pBR322 (comprising residues 241-1310) was prepared as described previously^31^.

### AFM methods

Topoisomerase enzymes at a concentration of ~200 nM were incubated with the 1070 bp DNA fragment at a ratio of ~2:1 for 15 mins by diluting in de-ionised water. These complexes were diluted further to a final DNA concentration of 1–2 nM in 10 µL of deposition buffer (4 mM Hepes (pH 7.4), 10 mM NaCl, 2 mM MgCl_2_) and deposited onto freshly cleaved mica. The sample was incubated for 60 s and then rinsed with excess de-ionised water and dried with a weak flux of nitrogen (exit pressure 1 bar). Topographical (Z-piezo) and phase contrast images were collected in ambient air with a Nanoscope IIIa AFM (Bruker, Santa Barbara, CA) at a relative humidity between 30 and 40%. AC160TS Cantilevers (Asylum Research) were used for dynamic AFM imaging having a length of 160 μm, and a measured spring constant of 40 N/m and a resonant frequency of 300 kHz. The tip radius is quoted by the manufacturer (Olympus) to be 9 ± 2 nm. High magnification scans of either 600 × 600 nm or 1 µm × 1 µm were collected with a slow scan frequency of 1.5 to 2.5 Hz at 512 × 512 pixel resolution.

AFM images were analysed using the Nanoscope AFM software. The centre of each protein was taken as the intersection of tangents defined for the incoming and outgoing DNA strands and the DNA bend angle was taken to be the angle inside these two tangents (see Figure 2). To determine the protein size in the AFM, the cross-section analysis tool was used and the diameter of the protein taken as the straight line distance from a threshold just above the background noise. Some of the topo proteins have an elongated appearance in the AFM images. To determine the protein diameter (D), measurements of the short and long axes were taken on up to 20 representative molecules for each topo protein, for both the symmetric and asymmetric complexes, and averaged. The diameters of other proteins imaged by AFM in previously published bend angle studies were measured directly from the grey-scale images in the publication.

## Author Contributions

N.H.T. and A.M. conceived the project and wrote the paper, SS carried out the experiments, L.A.M., T.S. and J.A.T. purified and provided the materials.

## Supplementary Material

Supplementary Information Supplementary Material

## Figures and Tables

**Table 1:** Summary of bend angle measurements for type II topoisomerases. The mean bend angle and standard deviation for all four enzymes are shown for the fits to both the full and split asymmetric distributions for n (number of molecules measured). The value for *S. shibatae* topo VI is from the lower peak in the fit of two folded Gaussians

Enzyme	Complexes measured: n	Mean bend angle of asymmetric complexes	S.D. of bend angle of asymmetric distribution	Number of asymmetric (bent) complexes: N_asym_	Number of symmetric (unbent) complexes: N_sym_	Ratio: N_asym_/N_sym_
*E. coli* topo IV	167	111.5	19.4	115	52	2.21
*M. mazeii* topo VI	147	120.3	23.8	86	61	1.41
*S. shibatae* topo VI	226	123.8	21.0	86	120	0.72
Yeast topo II	179	119.5	17.1	105	74	1.42
