# Supplementary material for: DNA G-segment bending is not the sole determinant of topology simplification by type II DNA topoisomerases
Source: Sci Rep. 2014 Aug 21;4:6158. doi: 10.1038/srep06158 (PMC4139952; doi:10.1038/srep06158)
Supplement: Supplementary Information — Supplementary Material [file srep06158-s1.pdf]

Supplementary Material

DNA G-segment bending is not the sole determinant of topology simplification by type II DNA topoisomerases

Neil H. Thomson[1], Sergio Santos[1,†], Lesley A. Mitchenall[2], Tanya Stuchinskaya[2,‡], James A. Taylor[2,¥], and Anthony Maxwell[2]

[1]Department of Oral Biology, School of Dentistry and Molecular and Nanoscale Physics Group, School of Physics and Astronomy, University of Leeds, Leeds, LS2 9JT, United Kingdom. [2]Department of Biological Chemistry, John Innes Centre Norwich Research Park, Norwich NR4 7UH, United Kingdom.

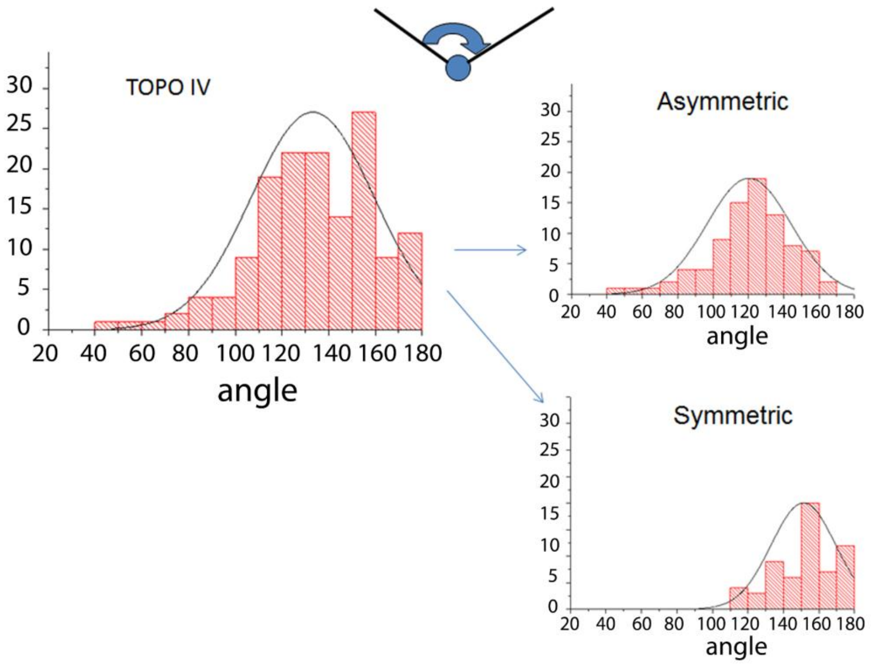

Figure S1 │ Bend angle distributions. The full bend angle distributions were split into symmetric and asymmetric complexes (i.e. bent and unbent) by visual inspection by two independent operators. This lowered the determined bend angles by 3.5 to 16.1° and the standard deviation decreased by 1.1 to 3.1°. Data shown is for *E. coli* topo IV.

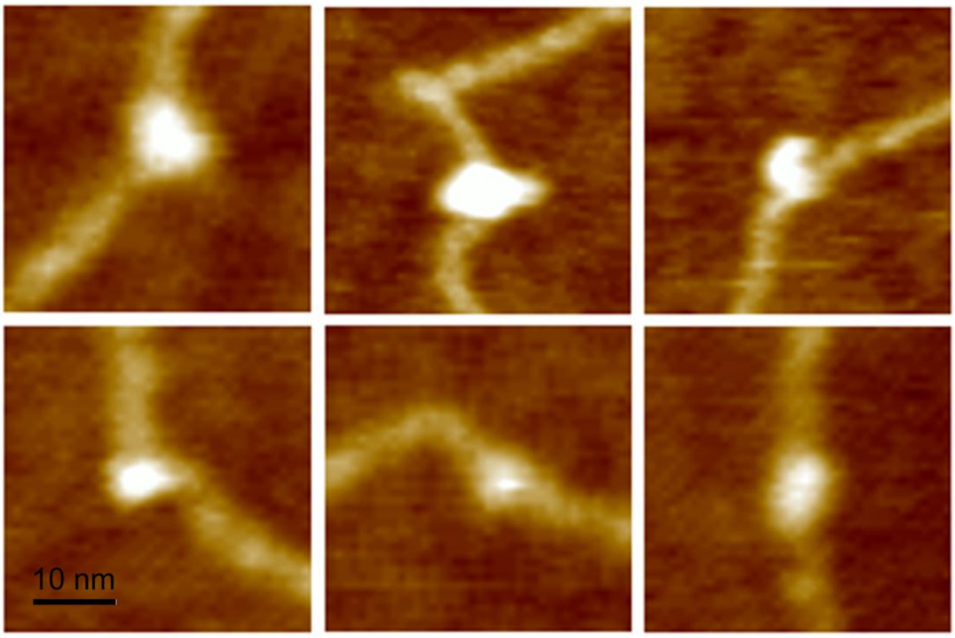

Figure S2 | Yeast topo II. Example SASS mode AFM topography images of Yeast topo II. These are software zooms taken from lower magnification images as detailed in the methods section.

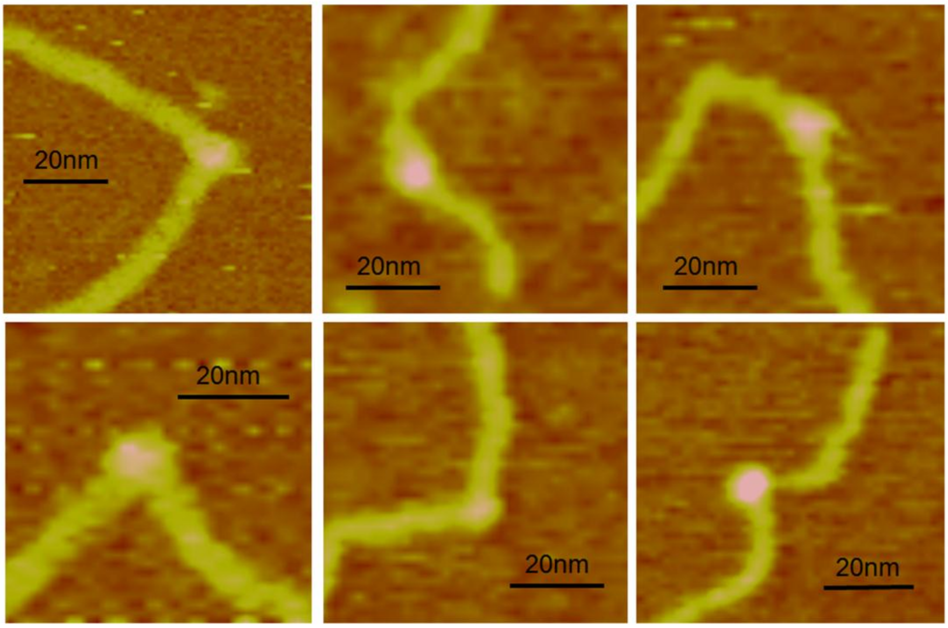

Figure S3 | *E. coli* topo IV. Example SASS mode AFM topography images of *E. coli* topo IV. These are software zooms taken from lower magnification images as detailed in the methods section.

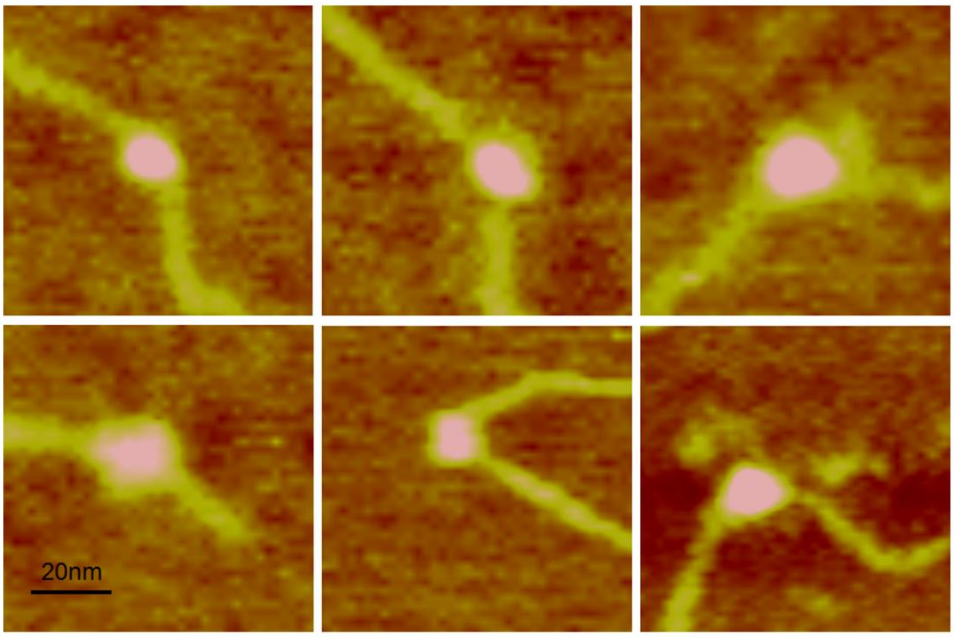

Figure S4 │ *M. mazei* topo VI. Example SASS mode AFM topography images of *M. mazei* topo VI. These are software zooms taken from lower magnification images as detailed in the methods section.

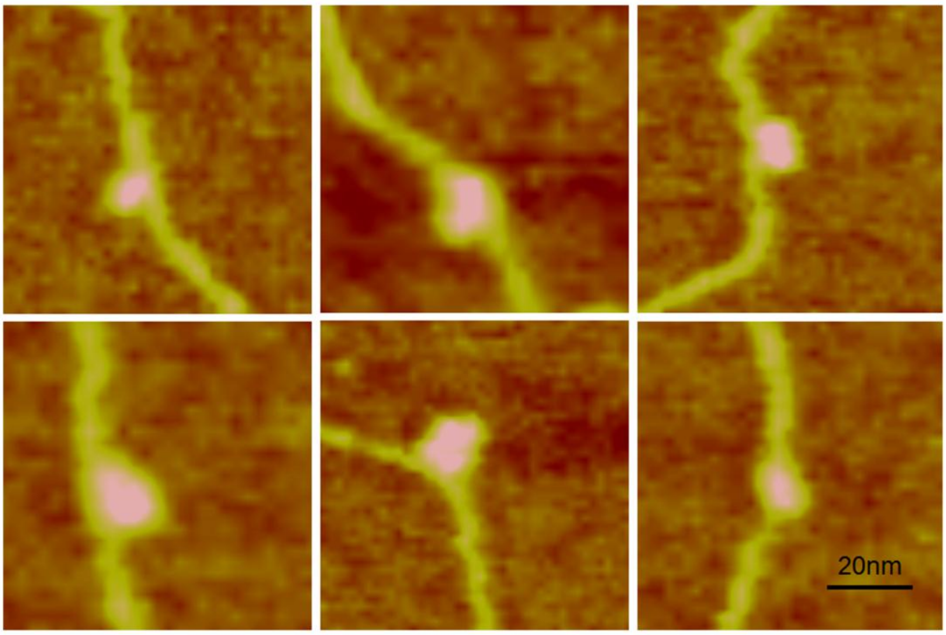

Figure S5│ *S. shibatae* topo VI. Example SASS mode AFM topography images of *S. shibatae* topo VI. These are software zooms taken from lower magnification images as detailed in the methods section.
